# Latent profile analysis of symptoms of depression and anxiety among perimenopausal women and their predictors

**DOI:** 10.3389/fpsyt.2025.1572570

**Published:** 2025-06-04

**Authors:** Minhui Jiang, Liang Chen, Xiaomin Zheng, Yaling Feng

**Affiliations:** ^1^ Department of Psychology, Affiliated Women’s Hospital of Jiangnan University, Wuxi, China; ^2^ Wuxi School of Medicine, Jiangnan University, Wuxi, China; ^3^ Mental Health Education Center, Zhejiang A&F University, Hangzhou, China; ^4^ Research Institute for Reproductive Health and Genetic Diseases, Wuxi Maternal and Child Health Hospital, Wuxi, China

**Keywords:** anxiety, depression, perimenopausal women, latent profile analysis, predictors

## Abstract

**Purpose:**

This study aims to consider the latent profiles of depression and anxiety symptoms in perimenopausal women and determine the influencing factors of this classification.

**Methods:**

Latent profile analysis was used to explore the subgroups of mental health in perimenopausal women (n=1242, age = 52.55 ± 3.32 years), followed by univariate analysis and multinomial logistic regression to investigate the relationship between sociodemographic, lifestyle, social interaction, perimenopausal symptom factors, and the latent profiles.

**Results:**

Depression and anxiety symptoms in perimenopausal women were classified into three subgroups: “low symptom group” (n=702, 56.5%), “depression and anxiety borderline symptom group”(n=419, 33.7%), and “severe depression and anxiety comorbidity group”(n=121, 9.8%). Additionally, empty nest, chronic diseases, history of mental illness, sleep quality, and perimenopausal symptoms were identified as risk factors for depression and anxiety, while enrolling in urban resident medical insurance and participating in social activities were protective factors.

**Conclusion:**

This study identified the heterogeneous characteristics of mental health in perimenopausal women to help provide more targeted screening, personalized interventions and treatment strategies, which are of significant importance in improving the quality of life for perimenopausal women.

## Introduction

Perimenopause is a transitional phase in middle-aged women that leads to reproductive aging, starting from irregular menstrual cycles and lasting up to 12 months after menopause (1). According to the World Health Organization (WHO), it is estimated that by 2030, the number of perimenopausal women will reach 1.2 billion, with nearly 76% residing in developing countries (2). With the trend of aging population in China, it is projected that by 2030, the number of menopausal women in China will increase to 280 million. Women often experience physical, psychological, sexual and behavioral symptoms during perimenopause (3), such as vasomotor symptoms, vaginal dryness, decreased libido, insomnia, fatigue, hot flashes, forgetfulness, mental symptoms (depression and anxiety), and decreased cognitive abilities (4). Perimenopause is a phase where women are more susceptible to anxiety and depression compared to premenopausal and postmenopausal women (5). The fluctuations in hormone levels, chronic diseases, and negative life events (e.g., retirement, marital discord, empty nest syndrome, rebellious adolescents, and caring for elderly parents) have been associated with increased vulnerability to mood disorders in perimenopausal women (6–8). It has even been proposed that “perimenopausal depression” is a unique subtype of depression, for which a specific assessment tool has been developed (9). Studies have reported that the prevalence of depression and anxiety symptoms in Chinese perimenopausal women ranges from 18.2% to 26.0% and 7.0% to 12.6%, respectively (10, 11). Furthermore, mood disorders during perimenopause have deleterious effects on women’s daily social functioning, quality of life, sleep quality, and life satisfaction (11–14). Therefore, perimenopausal period is considered a crucial time for understanding and addressing women’s mental health issues.

Depression and anxiety, recognized as two primary types of mental disorders, have emerged as global concerns for perimenopausal mental health (15). Studies have shown that depression often co-occurs with anxiety (16), and there is a correlation between the severity of anxiety and depressive symptoms (17). While certain studies have explored the prevalence and susceptibility factors of perimenopausal depression (5, 18–20), research on perimenopausal anxiety has been comparatively overlooked. Various studies lack standardized measures of anxiety (21), and the correlation between anxiety and perimenopausal symptoms has also been weak or even insignificant (22). Nonetheless, the importance of anxiety should not be ignored when exploring the relationship between perimenopausal and emotional symptoms. Additionally, much of the existing research has predominantly focused on the linear associations between the severity of perimenopausal anxiety and depressive symptoms, leaving our understanding of the underlying mechanisms behind the comorbidity of anxiety and depression in perimenopausal women still limited. Fried et al. (23) highlighted significant individual variance in symptom presentation, individuals with similar overall levels of anxiety or depression can exhibit markedly different patterns and characteristics of symptoms. Identifying subgroups with shared symptom profiles could facilitate more targeted screening, early detection, or support for individuals struggling with mental health. Latent profile analysis (LPA) is a person-centered approach used to identify latent subgroups with distinct symptom patterns (24). When examining the heterogeneous characteristics of mental health in perimenopausal women, the person-centered approach provides greater insights compared to a variable-centered approach. LPA has been extensively applied within the field of psychology (25).

Scholars have shown that individual subgroups exhibit co-morbid patterns and symptoms of depression and anxiety. Yu et al. utilized the LPA to identify four co-morbid patterns in the general older adult population, namely, “low depression and anxiety symptoms”, “mild depression only”, “moderate depression and anxiety” and “severe depression and anxiety”. Older adults who are male, sickly, and lack a healthy lifestyle are more likely to experience more severe mood symptoms (26). In another study, Zhou et al. categorized women aged 65 and older into three subgroups: “comorbidity depression and anxiety group”, “depression only group”, and “low symptom group”. They also discovered differences among the subgroups concerning age, place of residence, education level, income, assessments of current life and health status, sleep duration, and health behaviors such as alcohol consumption and exercise (27). However, there has not been any research utilizing LPA to explore the mental health of perimenopausal women. Therefore, it is essential to investigate the potential anxiety and depression comorbidity patterns in perimenopausal women in China and to identify the predictive factors of these patterns.

Anxiety and depressive symptoms in perimenopausal women are influenced by a variety of factors, including diabetes (28), history of depression (29), increased menopausal symptoms and sleep disorders (15, 30), and high body mass index (31). A previous study indicated that poor health, difficulty in initiating sleep, and early waking are associated with anxiety symptoms, while a higher BMI, poor health status, low educational attainment, and night sweats correlate with depressive symptoms (11). Furthermore, Wang et al. (32) showed that age, household income, regular physical activity, chronic illness, menopausal status, vasomotor symptoms, somatic symptoms, and urinary and reproductive system symptoms were related to depressive symptoms, whereas factors such as residence, regular exercise, chronic conditions, vasomotor issues, somatic complaints, and symptoms related to the urinary and reproductive systems were related with anxiety symptoms. However, there is a scarcity of studies examining the factors influencing the mental health of different subgroups of perimenopausal women. Previous research predominantly focused on individual-level determinants, encompassing physical health, lifestyle, and menopausal symptoms, overlooking the influence of social-level factors. Therefore, we introduced the Health Ecology Model (HEM) (33), which assumes that individual health is influenced by personal characteristics and interpersonal networks as well as the social environment. We will analyze the determinants of the classification of depression and anxiety symptoms through four dimensions: demographic characteristics, lifestyle, social interaction, and menopausal symptoms, to provide a multidimensional and multifactorial exploration of the influences on the mental health of perimenopausal women.

The present study used LPA to explore the heterogeneity of anxiety and depression comorbidity patterns in perimenopausal women and further identify the predictive factors. The goal is to enhance awareness of mental health issues during the perimenopausal period, assisting women in achieving early recognition, reducing fear, and seeking timely and accurate intervention and treatment.

## Material and methods

### Participants

This was a cross-sectional study conducted between January 2023 and June 2024 at the Menopause Clinic and Psychological Clinic of Wuxi Maternal and Child Health Hospital. Inclusion criteria consisted of married perimenopausal women aged 45-60, while exclusion criteria included individuals with severe physical illnesses, undergoing psychotropic medication, hysterectomy or oophorectomy, use of oral contraceptives, pregnancy, or hormone therapy. After excluding incomplete questionnaire responses, a total of 1242 women (age = 52.55 ± 3.32 years) were included in the analysis. This study was approved by the Ethics Review Committee of Wuxi Maternal and Child Health Hospital, and all participants provided written informed consent.

### Measurements

#### Hospital anxiety and depression scale

The Chinese version of the Hospital Anxiety and Depression Scale (HADS) (34, 35) was utilized to assess symptoms of anxiety and depression. HADS consists of 14 items: 7 items assessing depression and 7 items assessing anxiety. Items are rated on a (0-3) 4-point Likert scale. The maximum score for each subscale is 21. Scores ranging from 0 to 7 are considered normal, scores from 8 to 10 indicate borderline symptoms, and scores between 11 and 21 reflect the presence of positive symptoms of anxiety or depression (36). The Cronbach’s α coefficient for HADS in this study was 0.887.

#### Predictor variable factors

A structured questionnaire was used to assess potential influences on the mental health of perimenopausal women, categorized into four dimensions: Demographic characteristics, including age, marital status (married/divorced or widowed), residence (urban/rural), education level (primary or junior high school/high school/college or undergraduate), employment status (employed/unemployed), empty nest status (yes/no), average monthly household income (below 10,000 yuan/10,000-20,000 yuan/above 20,000 yuan), BMI (underweight/normal/overweight), urban resident medical insurance participation (yes/no), chronic illnesses (hypertension, diabetes, or dyslipidemia), and history of mental health disorders (depression/anxiety). Lifestyle factors included smoking (yes/no), alcohol consumption (yes/no), regular physical exercise (yes/no), intensity of physical activity (high/low), and sleep quality. Social interaction factors involved marital relationships (harmonious/strained), parent-child relationships (harmonious/strained), and participation in social activities (yes/no). Perimenopausal symptom factors include somatic symptoms, urogenital symptoms, and Kupperman Menopausal Index (KMI) scores.

The modified Chinese version of the Kupperman Menopausal Index (KMI) was utilized to evaluate the severity of menopausal symptoms (37). KMI consists of three symptom clusters: urogenital symptoms (such as urinary tract infections and sexual dysfunction), somatic symptoms (including sweating/hot flashes, palpitations, dizziness, headaches, abnormal skin sensations, sensory disturbances, joint pain, myalgia, insomnia, and fatigue), and psychological symptoms (irritability and depression). A four-point Likert scale (0-3) was employed to assess the severity of each item, with total scores ranging from 0 to 63. Symptoms were classified into four categories based on the total score: asymptomatic: <6, mild perimenopausal symptoms: 6–15, moderate perimenopausal symptoms: 16–30, severe perimenopausal symptoms: ≥31. Due to the overlap of items between the KMI and HADS, psychological items were excluded from the KMI for recalculating the total score. The Cronbach’s α coefficient for KMI in this study was found to be 0.737.

The Pittsburgh Sleep Quality Index (PSQI) was used to assess the sleep quality of the participants, which consists of 19 self-reported items and 5 other-reported items, including 7 factors, with higher scores indicating poorer sleep quality. Liu Xianchen et al. (38) have validated the Chinese version of this scale and verified that it has good psychometric properties. The Cronbach’s α coefficient for the 7 factors of the PSQI in this study was 0.522.

### Statistical analysis

Firstly, descriptive analysis was conducted on the total scores of depression and anxiety, as well as all predictor variables. Subsequently, Latent Profile Analysis (LPA) was performed using scores from all items on HADS to identify potential categories of depressive and anxiety symptoms among perimenopausal women. The analysis initiated with a one-class model and iteratively added to five classes to determine the best-fitting model. Model fit was evaluated using criteria such as the Akaike Information Criterion (AIC), Bayesian Information Criterion (BIC), sample-size adjusted BIC (aBIC), entropy, the bootstrap likelihood ratio test (BLRT), and the Lo-Mendell-Rubin adjusted likelihood ratio test (LMRT). Lower values of AIC, BIC, and aBIC indicate better model fit, while significant BLRT and LMRT results suggest that the k-class model provides a more effective fit to the data than the (k-1) class model. Higher entropy values reflect greater classification accuracy (39). An entropy value greater than 0.8 indicates good class separation (40). Model selection prioritizes the BIC and BLRT indices while also considering the interpretability of the latent profiles’ clinical significance (39). The posterior probabilities from the model were utilized to assign each participant to their most probable category (41). Finally, chi-square tests and analysis of variance (ANOVA) were employed to screen factors influencing the classification of latent factors. Variables that were statistically significant in univariate analysis were included in multinomial logistic regression to identify factors affecting the latent profiles. Prior to multinomial logistic regression analysis, we assessed the model for multicollinearity. The tolerance values for each independent variable were all well above 0.1, and the variance inflation factors (VIFs) were all below 10, indicating the absence of multicollinearity. LPA was conducted using Mplus 7.4, and SPSS 25.0 was used for univariate analyses and multinomial logistic regression.

## Results

### Identification of latent profiles of depression and anxiety in perimenopausal women

Using the 14 items of HADS as continuous observable variables, we explored models with 1 to 5 latent profiles (see **Table 1**). The results of the VLMR test indicated no significance in the four-class and five-class model, leading to the exclusion. The AIC, BIC, and a-BIC values consistently decreased with an increasing number of classes, showing a significant inflection point at the three-class model. The entropy values were all greater than 0.9. Both BLRT and VLMR for the two-class and three-class models were statistically significant (P<0.05). Considering the practical significance, interpretability, and conciseness of the model, the three-class model was chosen as the optimal model.

**Table 1:** Fit indices of the LPA models of HADS.

Number of classes	AIC	BIC	aBIC	BLRT	VLMR	Entropy	Number of persons in each class
1	43962.908	44106.393	44017.453	_	_	_	_
2	39546.539	39766.891	39630.304	<0.001	<0.001	0.924	825/417
3	38189.055	38486.274	38302.041	<0.001	<0.001	0.924	702/419/121
4	37527.044	37901.131	37669.250	<0.001	0.1218	0.951	671/276/179/116
5	36478.965	36929.919	36650.392	<0.001	0.0629	0.959	188/526/108/121/299

AIC, Akaike information criteria; BIC, Bayesian information criteria; aBIC, adjusted Bayesian information criterion; BLRT, Bootstrap likelihood ratio test; VLMR, Vuong-Lo-Mendell-Rubin test.

### Naming and characteristics of latent profiles of depression and anxiety in perimenopausal women

**Figure 1:**
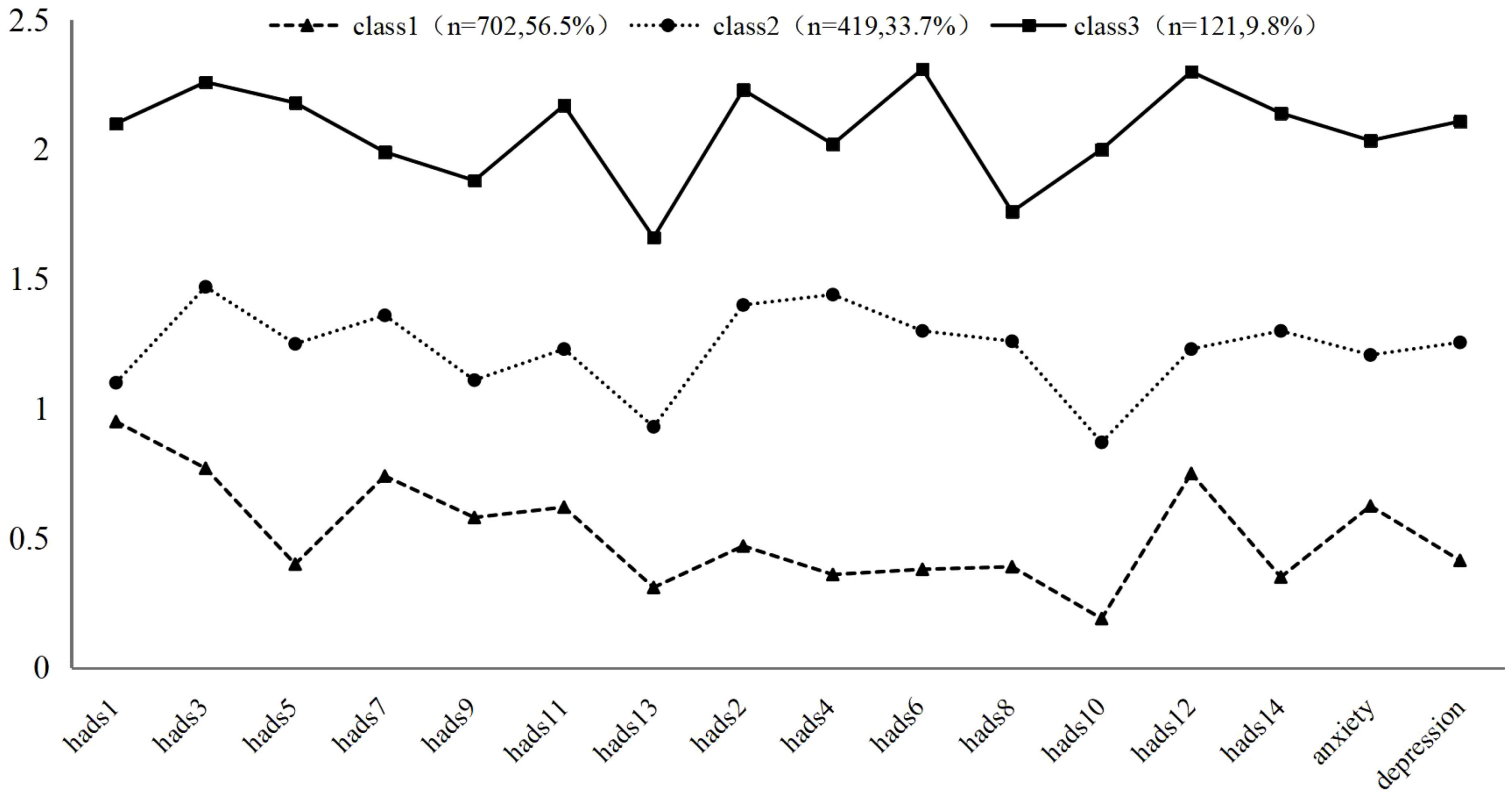
Latent profiles of HADS (N=1242). Class 1: Low symptom group, Class 2: Depression and anxiety borderline symptom group, Class 3: Severe depression and anxiety comorbidity group. Items of anxiety: hads1,3,5,7,9,11,13, items of depression: hads2,4,6,8,10,14.

Based on the results of the latent profiles, we illustrated the scores of three latent profiles on each item of HADS (see **Figure 1**). Class 1 (n=702, 56.5%) exhibited mean scores below 1 for all items, hence named the “low symptom group.” Class 2 (n=419, 33.7%) had mean scores ranging from 1 to 1.5, indicating borderline symptomatology for depression and anxiety, thus named the “depression and anxiety borderline symptom group.” Class 3 (n=121, 9.8%) had mean scores exceeding 2 for over half of the items, with item 6 “I feel cheerful” and item 12 “I seem to be getting more and more down in the dumps” showing mean scores above 3, significantly higher than the other two classes, and was therefore named the “severe depression and anxiety comorbidity group.”

### Predictive factors of each latent profile of depression and anxiety in perimenopausal women

Univariate analysis among latent profiles was analyzed, and the results from the chi-square test and ANOVA (see **Table 2**) indicated significant differences among participants in relation to factors such as empty nesting, enrollment in urban resident health insurance, chronic diseases, history of mental illness, sleep quality, marital relationships, parent-child relationships, participation in social activities, somatic symptoms, urogenital symptoms, and perimenopausal symptoms. However, no significant differences were found concerning variables like age, marital status, residence, educational level, employment status, average monthly household income, BMI, alcohol consumption, smoking, regular physical exercise, and physical activity intensity.

**Table 2:** Univariate analysis of demographic characteristics, lifestyle, social interactions, and perimenopausal symptom factors in relation to depression and anxiety subgroups of perimenopausal women.

Dimensions	Variables	Classification	Class1 (n=702, 56.5%)	Class2 (n=419, 33.7%)	Class3 (n=121, 9.8%)	χ^2^/F
Demographic characteristics	Age (Mean ± SD)		52.39 ± 3.32	52.81 ± 3.23	52.59 ± 3.62	2.09
	Marital status (n, %)	Married	687 (97.9)	408 (97.4)	120 (99.2)	1.44
		Divorced/widowed	15 (2.1)	11 (2.6)	1 (0.8)	
	Residence (n,%)	Rural	317 (45.2)	180 (43.0)	56 (46.3)	0.68
		Urban	385 (54.8)	239 (57.0)	65 (53.7)	
	Educational level (n,%)	Primary or junior high school	389 (55.4)	240 (57.3)	74 (61.2)	1.67
		High School	237 (33.8)	133 (31.7)	36 (29.8)	
		College or undergraduate	76 (10.8)	46 (11.0)	11 (9.1)	
	Employment status (n, %)	Employed	447 (63.7)	269 (64.2)	74 (61.2)	0.38
		Unemployed	255 (36.3)	150 (35.8)	47 (38.8)	
	Empty nests status (n,%)	Yes	177 (25.2)	137 (32.7)	42 (34.7)	9.58^**^
		No	525 (74.8)	282 (67.3)	79 (65.3)	
	Average monthly household income (n, %)	Below 10,000 yuan	217 (30.9)	144 (34.4)	46 (38.0)	4.59
		10000–20000 yuan	334 (47.6)	190 (45.3)	57 (47.1)	
		Above 20,000 yuan	151 (21.5)	85 (20.3)	18 (14.9)	
	BMI (n,%)	Underweight	134 (19.1)	77 (18.4)	18 (14.9)	1.49
		Normal	144 (20.5)	83 (19.8)	24 (19.8)	
		Overweight	424 (60.4)	259 (61.8)	79 (65.3)	
	Urban resident medical insurance (n,%)	Yes	579 (82.5)	323 (77.1)	86 (71.1)	10.61^**^
		No	123 (17.5)	96 (22.9)	35 (28.9)	
	Chronic diseases (hypertension, diabetes, hyperlipidemia, etc.) (n,%)	Yes	101 (14.4)	88 (21.0)	12 (9.9)	12.35^**^
		No	601 (85.6)	331 (79.0)	109 (90.1)	
	History of mental illness (depression/anxiety) (n,%)	Yes	29 (4.1)	38 (9.1)	18 (14.9)	23.60^***^
		No	673 (95.9)	381 (90.9)	103 (95.1)	
Lifestyle	Alcohol consumption (n,%)	Yes	55 (7.8)	23 (5.5)	11 (9.1)	2.92
		No	647 (92.2)	396 (94.5)	110 (90.9)	
	Smoking (n,%)	Yes	35 (5.0)	20 (4.8)	7 (5.8)	0.20
		No	667 (95.0)	399 (95.2)	114 (94.2)	
	Regular physical exercise (n,%)	Yes	80 (11.4)	48 (11.5)	17 (14.0)	0.73
		No	622 (88.6)	371 (88.5)	104 (86.0)	
	Intensity of physical activity (n, %)	High	261 (37.2)	151 (36.0)	43 (35.5)	0.22
		Low	441 (62.8)	268 (64.0)	78 (64.5)	
	Sleep quality (Mean ± SD)		3.46 ± 1.29	4.62 ± 1.59	8.21 ± 1.93	557.07^***^
Social interaction	Marital relationship (n,%)	Harmonious	678 (96.6)	394 (94.0)	109 (90.1)	10.84^**^
		Strained	24 (3.4)	25 (6.0)	12 (9.9)	
	Parent-child relationships (n,%)	Harmonious	670 (95.4)	400 (95.5)	108 (89.3)	8.57^*^
		Strained	32 (4.6)	19 (4.5)	13 (10.7)	
	Participation in social activities (n,%)	Yes	280 (39.9)	131 (31.3)	34 (28.1)	11.97^**^
		No	422 (60.1)	288 (68.7)	87 (71.9)	
Perimenopausal symptoms	Somatic symptoms (Mean ± SD)		7.30 ± 5.45	9.89 ± 6.31	12.45 ± 6.80	52.83^***^
	Urogenital symptoms Mean ± SD)		2.62 ± 2.25	3.26 ± 2.24	3.49 ± 1.99	15.32^***^
	Perimenopausal symptoms (KMI scores) (Mean ± SD)		9.92 ± 6.41	13.15 ± 7.08	15.94 ± 6.87	58.93^***^

^*^p< 0.05, ^**^p< 0.01, ^***^p< 0.001.

Using the “low symptom group” as a reference, the results of the unordered multinomial logistic regression (see **Table 3**) revealed statistically significant influencing factors, which included empty nester status, enrollment in urban resident health insurance, chronic diseases, history of mental illness, sleep quality, participation in social activities, and perimenopausal symptoms. We found that compared to the “low symptom group,” women with history of mental illness, sleep disorders, and severe perimenopausal symptoms had a likelihood of 2.058 times (95% CI: 1.184-3.577), 1.888 times (95% CI: 1.689-2.111), and 1.080 times (95% CI: 1.057-1.103), respectively, to belong to the “depression and anxiety borderline symptom group.” Their likelihood of being categorized as the “severe depression and anxiety comorbidity group” was 3.677 times (95% CI: 1.305-10.363), 7.188 times (95% CI: 5.472-9.443), and 1.146 times (95% CI: 1.093-1.202), respectively. Additionally, women who actively participated in social activities reduced their chances of being in the “depression and anxiety borderline symptom group” and “severe depression and anxiety comorbidity group” (OR=0.340-0.683). Furthermore, women who were empty nesters and those with chronic diseases were 1.401 times (95% CI: 1.017-1.929) and 1.711 times (95% CI: 1.196-2.448) more likely to belong to the “depression and anxiety borderline symptom group”, while those with urban resident health insurance were less likely to be classified in the “depression and anxiety borderline symptom group” (OR=0.688).

**Table 3:** Multinomial logistic regression analysis of demographic characteristics, lifestyle, social interaction and perimenopausal symptom factors on depression and anxiety subgroups of perimenopausal women.

Predictor variables	Depression and anxiety borderline symptom group (n=419,33.7%)		Severe depression and anxiety comorbidity group (n=121,9.8%)	
	OR	95% CI	OR	95% CI
Empty nest: yes vs. no	1.401^*^	1.017-1.929	1.104	0.536-2.274
Enrollment in urban resident health insurance: yes vs. no	0.688^*^	0.485-0.977	0.675	0.315-1.447
Chronic diseases: yes vs. no	1.711^**^	1.196-2.448	0.621	0.225-1.719
History of mental illness: yes vs. no	2.058^*^	1.184-3.577	3.677^*^	1.305-10.363
Marital relations: harmonious vs. strained	0.691	0.346-1.380	0.824	0.188-3.615
Parent-child relationships: harmonious vs. strained	0.894	0.448-1.784	0.446	0.087-2.286
Participation in social activities: yes vs. no	0.683^*^	0.503-0.927	0.340^**^	0.153-0.755
Sleep quality	1.888^***^	1.689-2.111	7.188^***^	5.472-9.443
Perimenopausal symptoms	1.080^***^	1.057-1.103	1.146^***^	1.093-1.202

^*^p< 0.05, ^**^p< 0.01, ^***^p< 0.001.

## Discussion

Our study identified three subgroups of anxiety and depression among perimenopausal women. Specifically, 56.5% of participants demonstrated minimal levels of depression and anxiety that did not meet clinical diagnostic criteria, categorizing them as the low symptom group. Meanwhile, 33.7% exhibited borderline symptoms of anxiety and depression, classifying them as the “depression and anxiety borderline symptom group.” A smaller portion, 9.8%, reported more severe anxiety and depressive symptoms, indicating that anxiety and depression were comorbid (42, 43), thereby belonging to the “severe depression and anxiety comorbidity group.” Several studies on elderly populations using LPA have also identified “depression and anxiety comorbidity group” and “low symptom group” (26, 27), with distribution patterns similar to those observed in perimenopausal women of our research. Notably, although the “depression only group” was not identified in our study, the risk of depression in the moderate to severe symptom group was more pronounced than anxiety. This supports the findings of numerous studies that the incidence of depressive symptoms is higher than that of anxiety symptoms in perimenopausal women (11, 32, 44). In addition, the “severe depression and anxiety comorbidity group” is the most important group to focus on. The group scored significantly higher on depression negative item 6 “I feel happy” and depression positive item 12 “I seem to be getting more and more depressed,” indicating more prominent depressive symptoms, which often lead to poor quality of life and even suicidal ideation (45). Therefore, the prevention and intervention of anxiety and depression among perimenopausal women in China deserve high attention.

Our study also identified predictive factors associated with latent subgroups of anxiety and depression in perimenopausal women, including empty nest syndrome, urban resident medical insurance, chronic diseases, history of mental illness, sleep quality, participation in social activities, and perimenopausal symptoms.

In terms of demographic characteristics, perimenopausal women with history of mental illness were faced an increased risk of moderate to severe depression and anxiety, further validating previous research (29, 46). Various studies have emphasized that a history of depression predicts an increase in depressive symptoms and even severe major depression among perimenopausal women, with those having a history of mental illness tending to experience more intense perimenopausal symptoms (15, 29, 46). Therefore, it is crucial to screen the mental health status of perimenopausal women and to implement educational and preventive strategies that promote good mental health. Additionally, perimenopausal women with chronic diseases have a heightened likelihood of exhibiting “depression and anxiety borderline symptom group,” aligning with previous findings (28, 32). Metabolic syndromes such as hypertension, diabetes, and dyslipidemia, which are common health issues among middle-aged individuals, burden women not only with ongoing chronic diseases but also with the inevitable challenges of aging. This dual psychological and physiological strain exacerbates the loss of individual self-worth among middle-aged women, leading to emotional instability. This suggests the necessity for the continuous enhancement of public health service systems, with a focus on early screening and management of chronic diseases among perimenopausal women. Furthermore, the empty nest phenomenon also heightens the risk of depression and anxiety in perimenopausal women. The empty nest state exacerbates emotional vulnerability and psychological stress through weakened family support and increased loneliness. Chinese women in the perimenopausal stage may encounter pressures from diverse lifestyles and family transitions, including financial strain, the responsibilities of caring for elderly parents, rebellious adolescents, marital challenges, and the empty nest syndrome resulting from children leaving home. Cultural factors in China may also influence the mental health of perimenopausal women, for instance, women are often assigned greater familial responsibilities for nurturing and child-rearing compared to men, and Chinese parents frequently experience the financial stress related to purchasing new homes for their children during this stage. The study by Nagda et al. (44) also demonstrated a significant correlation between psychosocial stressors and the severity of anxiety and depression among perimenopausal women. Just as de Kruif et al. (47) noted, “The transitional phase of menopause can be seen as the last straw that breaks the camel’s back.” Furthermore, enrollment in urban resident medical insurance can reduce the risk of perimenopausal women developing the borderline symptoms of anxiety and depression. This finding is consistent with previous research (10), suggesting that improved medical insurance and pension plans can alleviate economic stress and enhance quality of life, consequently reducing the incidence of depression and anxiety. Evidence indicated that medical assistance and healthcare services can also buffer older adults from mental problems (48). Therefore, it is imperative to enhance socioeconomic welfare services for perimenopausal women and elderly adults, such as advocating for universal urban resident medical insurance program, reinforcing government social security measures, and establishing comprehensive pension systems, thus further alleviating the challenges posed by China’s aging population as well as the associated risks of depression and anxiety.

In terms of lifestyle, women with sleep quality problems exhibited increased risk of moderate to severe depression and anxiety, aligning with the findings of numerous previous studies (11, 30, 49–51). Sleep disturbances are prevalent among individuals suffering from depression and those undergoing menopause, significantly impacting the physical and mental health of perimenopausal women (52). Several studies suggested that the increase in sleep problems among middle-aged women may trigger depressive trajectories or escalate symptoms (30, 50, 53). while other research indicated that, compared to depression, poor sleep quality in perimenopausal women had a stronger relationship with anxiety (51). Women with sleep problems were approximately three times more likely to report anxiety symptoms than those without such problems (11). de Zambotti et al. (49) showed that insomnia symptoms in perimenopausal women were significantly associated with anxiety and depression. It can be seen that insomnia and sleep problems exacerbate emotional dysregulation through physiological and psychological interactions, negatively impacting quality of life and work efficiency (54). Thus, this study proposes Cognitive Behavioral Therapy (CBT) for insomnia in perimenopausal women, alongside necessary pharmacological sleep aids, including sleep hygiene education, relaxation therapy, stimulus control, sleep restriction, and cognitive therapy to assist them in better understanding and effectively managing their sleep problems.

In terms of social interaction, women who actively engage in social activities experienced reduced risk of moderate to severe depression and anxiety. This finding aligns with the study examining the factors influencing depression and anxiety in older adults (55). This is attributed to the fact that perimenopausal women participating in social activities can establish new social connections or reinforce existing interpersonal relationships, thereby enhancing their ability to adapt and cope with changes in environmental and social relationships. They can also obtain sufficient social support and experience positive emotions such as a sense of belonging and self-worth. Participation in social activities, as a form of behavioral activation, serves as an intervention to enhance interest and derive pleasure, proving to be an effective attempt to treat depression and menopausal symptoms (56). This highlights the necessity for community organizations to diversify social and recreational activities, helping perimenopausal women proactively engage in social activities.

In terms of perimenopausal symptoms, these symptoms served as significant risk factors for moderate to severe depression and anxiety, consistent with previous findings (10, 32, 57–61). The top five perimenopausal symptoms ranked by severity include insomnia, fatigue, musculoskeletal pain, sexual dysfunction, and emotional instability (62). Recent cross-sectional studies conducted on middle-aged women in China have discovered positive correlations between vasomotor symptoms, somatic symptoms, and urogenital symptoms with both depression and anxiety symptoms (10, 32). Several studies have found significant correlations between menopausal symptoms and depressive symptoms (58, 60, 61), other studies have found that menopausal symptoms not only partially mediate the relationship between lifestyle factors and depressive symptoms (63), but can also significantly influence depression by promoting healthier lifestyles (64). Additionally, two longitudinal studies have supported the notion that menopausal symptoms are closely related to anxiety symptoms (57, 59). This association may be partly attributed to the decline in estrogen levels, leading to increased serum monoamine oxidase activity, which reduces serum serotonin concentrations, thereby inducing or exacerbating mood disorders. Alternatively, it could stem from neuroendocrine dysfunction triggered by hormonal changes, heightening women’s sensitivity and vulnerability to physiological, psychological, social, and environmental factors, consequently resulting in emotional instability, psychosomatic dysfunction, and diminished quality of life (65). Consequently, Hormone Replacement Therapy (HRT) is considered as a vital treatment approach for alleviating perimenopausal symptoms. Educating women on the importance of HRT and enhancing their acceptance and utilization of HRT may prevent or reduce the incidence of perimenopausal syndrome and mood disorders (15, 66).

In summary, this study is the first to employ LPA to explore the patterns and predictive factors of anxiety and depressive symptoms among perimenopausal women in China. The findings can provide a more nuanced understanding of individual differences in perimenopausal women’s mood and contribute to personalized intervention and treatment strategies, which are important for improving the quality of life of perimenopausal women. This study emphasizes the importance of implementing comprehensive intervention measures, including encouraging perimenopausal mental health screening, universal social security, active participation in social activities, sleep guidance, chronic disease management, hormone replacement therapy, and cognitive behavioral therapy, aimed at reducing the risks of anxiety and depression, thereby helping them pass through the perimenopausal period safely and smoothly.

## Strengths, limitations and further research

Our study has several strengths. First, from an individual-centered perspective, we employed LPA to investigate the classification and comorbidity patterns of anxiety and depression among perimenopausal women, revealing a clustering characteristic in their mental health status. Furthermore, under the guidance of HEM, our study identified risk factors for depression and anxiety, including empty nest status, chronic illnesses, history of mental health disorders, sleep quality, and perimenopausal symptoms. Conversely, factors such as enrolling in urban resident health insurance and participation in social activities were found to be protective. Healthcare providers can utilize these identified patterns of anxiety and depression across different subgroups to develop personalized treatment and management strategies, thereby optimizing the allocation of healthcare resources.

However, this study also has its limitations. First, the data in this study came from a convenience sample of menopause clinics and psychological outpatient clinics in one city in China, and most of the participants had relatively obvious symptoms, which may limit the generalizability of the findings to non-clinical or other cultural populations. Given that geographical and cultural factors can influence symptoms of depression and anxiety, future research needs to conduct multi-center national studies across diverse regions to verify the accuracy of this pattern and ensure more diverse and representative samples. Second, this study employed a cross-sectional design that did not assess the causal relationships between depressive and anxiety symptoms and their associated factors. Future research should adopt longitudinal designs to verify whether our identified symptom patterns exhibit temporal stability and clarify the potential causal pathways between various predictive factors and perimenopausal emotional symptoms. Meanwhile, CBT and HRT intervention studies can be used to explore changes in perimenopausal depression and anxiety symptoms in the future. Third, as the co-occurrence of depressive and anxiety symptoms with menopausal symptoms can be explained by biological factors (67), future research could incorporate multidimensional data such as hormone levels, neural circuit, genetic, and molecular levels to further elucidate the underlying mechanisms of perimenopausal depression and anxiety (68). Finally, using self-reports to evaluate psychosocial variables introduced potential response bias. HADS served merely as a self-assessment tool for emotional issues and may not effectively distinguish between symptoms of anxiety and depression (69, 70). Future research might employ tools such as the Patient Health Questionnaire (PHQ-9) and Generalized Anxiety Disorder Scale (GAD-7) to more accurately differentiate depressive and anxiety symptoms for validating the class model of this study, while considering network analysis to explore the association patterns between anxiety and depressive symptoms.

## Conclusion

The current findings highlight the heterogeneous characteristics of perimenopausal women’s mental health in order to provide more targeted screening, personalized interventions, and treatment strategies. This study also emphasizes comprehensive interventions, including encouraging perimenopausal mental health screening, universal social security, active participation in social activities, sleep guidance, chronic disease management, hormone replacement therapy, and cognitive behavioral therapy to reduce the risk of anxiety and depression among perimenopausal women, thereby help them pass through the perimenopausal period safely and smoothly.

## Data Availability

The raw data supporting the conclusions of this article will be made available by the authors, without undue reservation.
